# Small Bowel Lymphangiomatosis Presenting With Amyloidosis: A Case Report

**DOI:** 10.7759/cureus.114498

**Published:** 2026-08-13

**Authors:** Garo J Naljian, Rachna P Patel, Tamaya S Mcqueary, Salvatore Grasso, Jian-Hua Qiao

**Affiliations:** 1 Emergency Medicine, Ross University School of Medicine, Los Angeles, USA; 2 Internal Medicine, Ross University School of Medicine, Los Angeles, USA; 3 Medicine, Ross University School of Medicine, Los Angeles, USA; 4 Pathology, California Hospital Medical Center, Los Angeles, USA

**Keywords:** aa amyloidosis, amyloidosis, amyloid plaques, intestinal amyloidosis, intestinal lymphangioma, lymphangioma, sap, serum amyloid protein

## Abstract

Small bowel lymphangiomatosis is a rare benign lymphatic malformation with nonspecific clinical manifestations that may complicate preoperative diagnosis. We present the case of a 57-year-old male with a history of recurrent umbilical hernias who complained of abdominal pain, nausea, vomiting, and an unreducible ventral hernia, all of which suggested a small bowel obstruction. Imaging revealed a presumed strangulated hernia with bowel obstruction and abnormal thickening of various loops of small bowel. Exploratory laparotomy disclosed incarcerated and gangrenous small bowel requiring resection. Histopathologic evaluation demonstrated extensive small-bowel lymphangiomatosis with concurrent AA-dominant amyloidosis, confirmed by apple-green birefringence on polarized microscopy of a Congo red-stained sample. This case highlights a rare coexistence of lymphangiomatosis and amyloidosis and suggests a possible relationship between chronic inflammatory amyloid deposition and secondary lymphatic obstruction, which may contribute to lymphangioma formation.

## Introduction

Lymphangiomas are rare, benign lesions of the lymphatic system that can arise throughout the skin and mucous membranes of the body. They are classified as either deep, including cavernous lymphangiomas and cystic hygromas, or superficial, including lymphangioma circumscriptum and acquired lymphangiomas. These two superficial lymphangiomas demonstrate the same clinical and histologic features but differ in etiology. Lymphangioma circumscriptum results from a congenital malformation of the lymphatic system, whereas acquired lymphangiomas, also called lymphangiectasias, result from external obstruction that dilates previously healthy lymphatic vessels [1]. A majority of congenital lymphatic malformations (approximately 70%) are in the head and neck because of the extensive lymphatic development in the area [2].

Lymphatic malformations represent 12% of all vascular malformations and 25% of all benign pediatric vascular tumors [1,3]. Acquired lymphangiomas are usually found in the axillary, inguinal, and genital areas, with coexisting lymphedema, and present with pain, itching, drainage, and/or infection. The most common locations for lymphangiomas of the intra-abdominal cavity are the mesentery, omentum, mesocolon, and retroperitoneum [4]. Small bowel lymphangiomas are extremely rare.

Diagnostic evaluation typically involves imaging modalities such as ultrasonography (U/S), computed tomography (CT), and magnetic resonance imaging (MRI), which aid in lesion localization and characterization [5]. Endoscopic evaluation can help in identifying gastrointestinal involvement. Definitive diagnosis is established through histopathologic examination. Management depends on lesion size, location, and symptom severity, with complete surgical excision considered the treatment of choice given the risk of recurrence with incomplete resection.

AA amyloid, a protein fragment derived from serum amyloid A (SAA), has an unfortunate tendency to deposit in tissues, leading to AA amyloidosis, a complication of chronic inflammation [6]. SAA is an acute-phase protein that’s highly involved in immune functions, lipid metabolism, and tissue remodeling and integrity. Although these insoluble fibers are primarily found in the kidney, gastrointestinal tract, and other supporting abdominal organs, they are often implicated in systemic conditions such as rheumatoid diseases, autoinflammatory syndromes, tuberculosis, inflammatory bowel disease, malignancies, and more [7]. Once biopsied and confirmed, revealing apple-green birefringence under polarized light on Congo-red stain, treatment is tailored to the underlying inflammatory issue. AP amyloid, on the other hand, is a plasma glycoprotein that is involved in all amyloid deposition regardless of the type of amyloidosis [8].

We present the case of a patient with a dual presentation of lymphangiomatosis and amyloidosis in the small bowel. While both pathologies are uncommon, having a combined presentation within the small bowel is exceedingly rare. Thus, their combined presentation has been poorly characterized. We hypothesize that this dual presentation developed secondary to a persistent inflammatory state caused by the lymphangiomatosis. This chronic inflammation likely precipitated the deposition of acute-phase proteins, which over time developed into the secondary amyloidosis. While alternative etiologies exist, our discussion will focus on this proposed pathophysiologic mechanism.

This work was previously presented as a poster presentation at California Hospital Medical Center on May 28^th^, 2026.

## Case presentation

Our patient is a 57-year-old male with a past medical history of recurrent umbilical hernias, obesity, hypertension, and type 2 diabetes who presented to the emergency room with abdominal pain, nausea, and vomiting. The patient reported repeated episodes of umbilical hernias, all of which had been reducible and had not required intervention. He had been feeling constipated for the past 5 days but had been passing small bowel movements (with use of enemas) and flatus, with the last episode being one day before the emergency room visit. Unlike his prior hernias, this episode had remained unreducible and persisted with gradually escalating symptoms. The patient denied any recent acute or chronic infections or other inflammatory pathologies.

After an initial evaluation, the patient was placed on NPO status with an NG tube in place and given fluids. Initial labs showed leucocytosis with a white blood cell count of 12.1x10^3^/mm^3 ^(reference range, 4.0-11.0x10^3^/mm^3^) and lactic acidosis with a lactic acid value of 2.8 mmol/L (reference range, 0.5-2.2 mmol/L). Acute kidney injury (AKI) was also noted with a significant blood urea nitrogen of 67 mg/dL (reference range, 7-20 mg/dL) and a creatinine of 1.7 mg/dL (reference range, 0.7-1.3 mg/dL), which gradually resolved with the help of intravenous fluids. This episode of AKI was diagnosed as prerenal azotemia secondary to excessive fluid loss due to dehydration and vomiting prior to the patient presenting to the emergency department.

**Figure 1:**
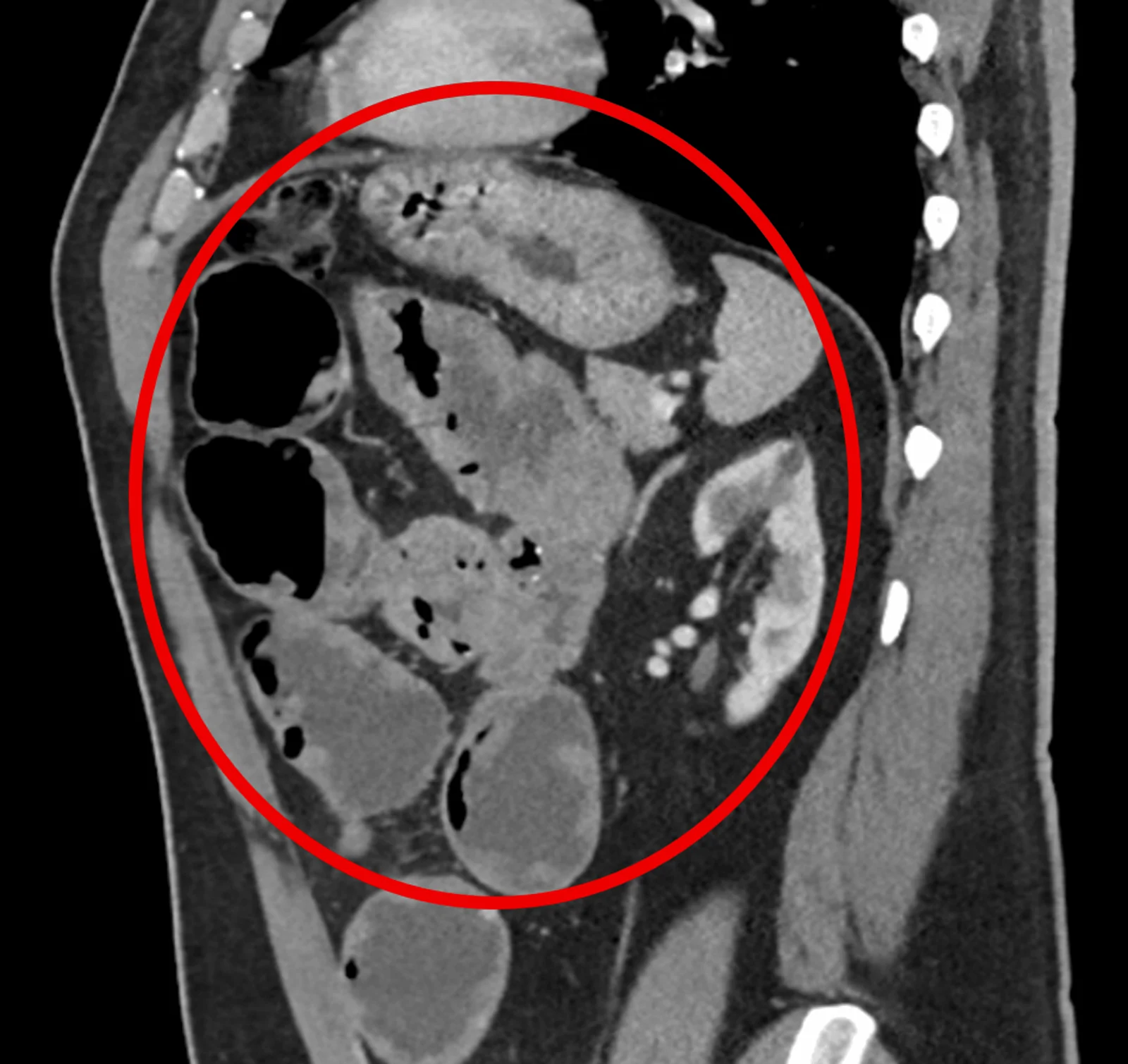
Sagittal CT abdomen without contrast showing segments of small bowel with thickened bowel walls.

CT scan of the abdomen without contrast showed a periumbilical hernia measuring 6.7 cm in maximal diameter with strangulation, as well as a proximal to mid small bowel obstruction (SBO) measuring up to 5 cm (Figure 1). Multiple loops of thickened small bowel walls were also visualized, morphologically suggesting a small bowel lymphangiomatosis. This was a prospective imaging diagnosis after the resected specimen was described as edematous and gangrenous-appearing during gross intraoperative examination and was also histologically confirmed.

The patient was then admitted to the medical-surgical unit, where his medical management was continued. Upon repeat imaging, the SBO persisted, and the patient was referred to the surgical team, who performed a laparotomy. The patient was noted to have an umbilical hernia with a small amount of omentum adhering to the hernia. Additionally, a large amount of adipose tissue was found to have a second ventral hernia with incarcerated small bowel. The incarceration had led to gross findings that were suggestive of gangrenous development of the loop of small bowel. This area was resected, and a side-to-side anastomosis was performed. The umbilical hernia was also repaired.

Postoperatively, the patient was monitored in the telemetry unit, where he received postoperative antibiotics along with pain management. Secondary to ileus, peripheral parenteral feeding was initiated but was shortly discontinued, and his diet was advanced as the ileus resolved.

Although the patient was discharged and advised to follow up with his primary care provider and surgical team, details of the follow-ups were not available. Additionally, he was prescribed pantoprazole, docusate, and acetaminophen-hydrocodone for at-home management and symptomatic control. 

**Figure 2:**
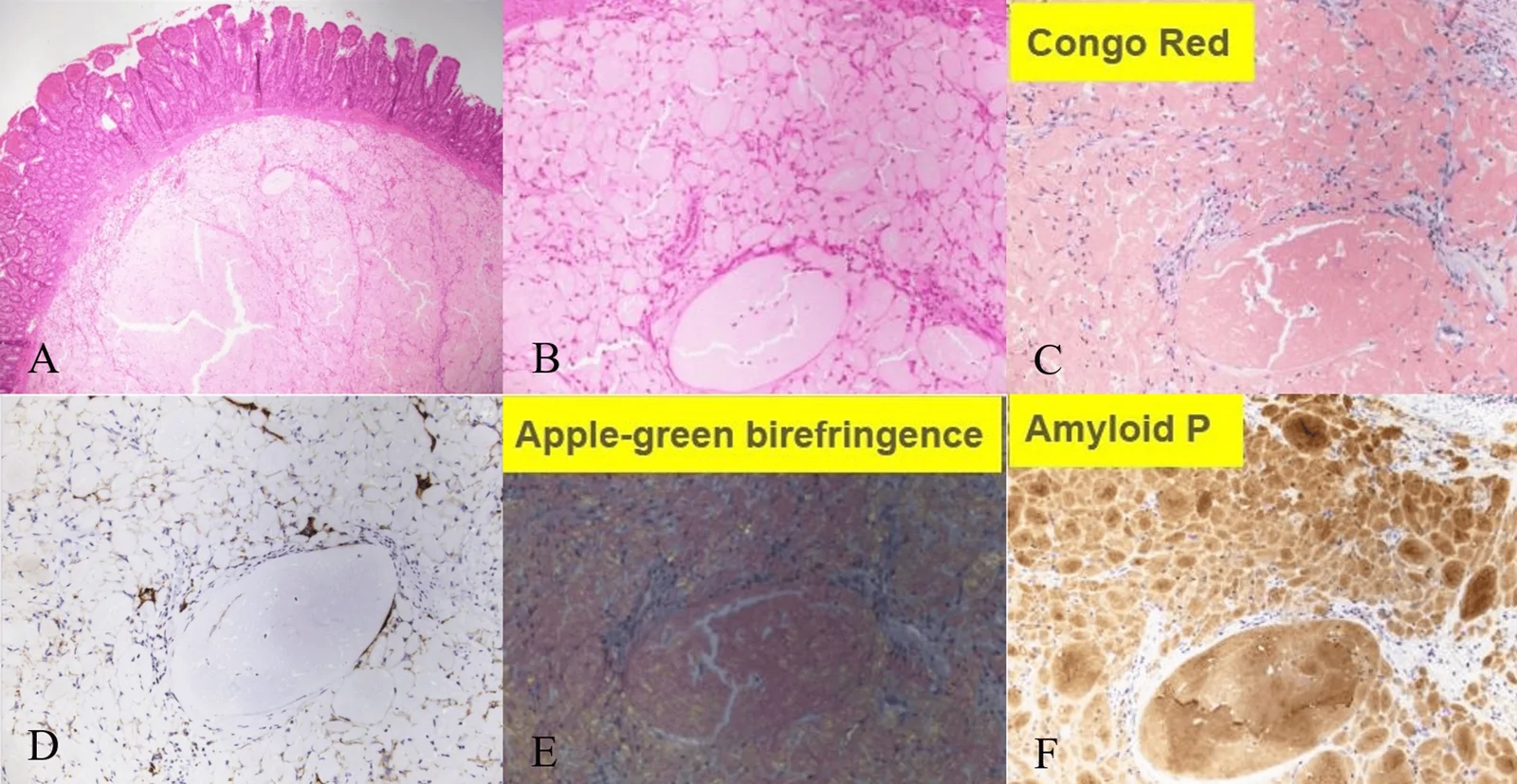
Histopathology findings A) Small bowel pseudopolyp with extensive submucosal lymphangiomatosis (H&E stain, 40X), B) Histologic image of amyloid deposition, C) Submucosal lymphangiomatosis with amyloidosis (H&E; Congo Red stain; IHC stain, 200X), D) D2-40 (IHC stain, 200X) highlights lymphangiomatosis, E) Apple-green birefringence by polarized microscopic examination of Congo-red stain (IHC stain, 200X), F) Amyloid P (IHC stain, 200X)

Microscopic examination of the resected small bowel revealed numerous submucosal crowded, dilated lymphatics, consistent with small bowel lymphangiomatosis involving the submucosa and the muscularis propria (Figure 2D). The fluid was thick and proteinaceous, raising suspicion of amyloidosis. Congo red staining observed under polarized microscopy revealed apple-green birefringence (Figure 2E), confirming the diagnosis of amyloidosis. Immunohistochemical stains for amyloid AA and AP were positive (Figure 2F). Additionally, areas within the mucosa were noted to contain pseudopolyps along with areas of ulceration (Figure 2A).

## Discussion

The coexistence of small bowel lymphangiomatosis and AA-dominant amyloidosis represents an unusual and rarely reported clinical finding. Acquired lymphangiomas resulting from lymphatic obstruction raise questions about a possible association between lymphatic abnormalities and conditions involving chronic inflammatory protein deposition, such as amyloidosis [9].

While lymphangiomas are generally considered congenital malformations of the lymphatic system, acquired lymphatic abnormalities may develop secondary to chronic obstruction or inflammation of previously normal lymphatic channels. Lymphangiomas tend to affect only the areas where they begin to develop and, as they grow, gradually lead to symptomatic presentations due to their disruption of surrounding systems [10]. Clinically, a typical presentation of a lymphangioma is a soft, mobile mass that is commonly non-tender. Histologic identification of lymphangiomas (Figure 2D) typically reveals dilated sacs lined by a single layer of endothelium [2]. These small bowel lymphangiomas also thicken the small bowel wall, making it more rigid. Continued thickening can lead to mucosal prolapse, forming pseudopolyps that further obstruct the lumen, as seen in our patient (Figure 2A).

Secondary to their inflammatory effects, some speculate that lymphatic disorders, including lymphangiomatosis, can lead to amyloidosis involving high amounts of both serum amyloid A and, with time, serum amyloid P (SAP) [9]. As a reminder, SAP is usually involved in all chronic amyloid deposits; thus, it isn’t unique to this case. It is important to consider the patient’s history of recurrent umbilical hernias, which may have developed secondary to the mass effect caused by the lymphangioma and its extensive inflammatory process. Depending on the extent of the thickening of the small bowel wall, the recurring herniations could eventually lead to the incarceration seen in this patient. The presence of thick proteinaceous intraluminal fluid, diffuse submucosal and muscularis propria involvement, and concurrent amyloid deposition further supports a pathophysiologic relationship between these processes. Due to these recurrent symptomatic presentations, surgical resection is commonly recommended for localized lesions. However, postoperative recurrence is common [10]. Other monosymptomatic patients are only observed, depending on the location and characteristics of the lymphangioma [11].

Although these disease processes may occur independently, their coexistence in the intestinal wall is unlikely to represent an isolated coincidence and more likely reflects an unusual and rare underlying pathophysiologic association.

## Conclusions

Our case highlights the rarity of small bowel lymphangiomatosis with AA-dominant amyloidosis in a patient with an incarcerated ventral hernia and small bowel obstruction. The coexistence of both pathologies suggests that chronic inflammatory changes and amyloid deposition may develop secondary to lymphatic channel obstruction and subsequent lymphangioma formation. Given the nonspecific clinical presentation and overall rarity of small bowel lymphangiomatosis, histopathologic evaluation remains essential for a definitive diagnosis. Given that this is a single case presentation, our suggested pathophysiologic mechanism requires further study to better characterize the relationship between amyloidosis and lymphatic abnormalities within the gastrointestinal tract. 

As a single case report, the findings from this patient cannot be generalized to the entire patient population. Although histologic means were used to confirm the diagnosis, mass spectrometry was not performed. Also, patient follow-up was not available after the initial presentation. However, for future cases, further evaluation for serum paraprotein, monoclonal gammopathy of undetermined significance (MGUS), and possible plasma cell myeloma is recommended.
